# Determinants of out-of-pocket health expenditure on children: an analysis of the 2004 Pelotas Birth Cohort

**DOI:** 10.1186/s12939-015-0180-0

**Published:** 2015-06-09

**Authors:** Marcelo Torres da Silva, Aluísio J. D. Barros, Andréa D. Bertoldi, Paulo de Andrade Jacinto, Alicia Matijasevich, Iná S. Santos, Cesar Augusto Oviedo Tejada

**Affiliations:** Programa de Pós-Graduação em Organizações e Mercados, Universidade Federal de Pelotas, Rua Gomes Carneiro 1, Pelotas, RS 96010-610 Brazil; Programa de Pós-Graduação em Epidemiologia, Universidade Federal de Pelotas, Pelotas, Brazil; Programa de Pós-Graduação em Economia do Desenvolvimento, Pontifícia Universidade Católica do Rio Grande do Sul, Porto Alegre, Brazil; Departamento de Medicina Preventiva, Faculdade de Medicina, Universidade de São Paulo, São Paulo, Brazil

**Keywords:** Determinants, Health expenditure, Children, Health economics, Inequality

## Abstract

**Background:**

The present study aimed to examine the impact of socioeconomic, demographic, and health status-related factors on out-of-pocket expenditure on health care for children.

**Methods:**

Data were obtained from a birth cohort study conducted in the city of Pelotas, state of Rio Grande do Sul (RS), southern Brazil, in 2004. The final sample is a result of adjusts made in order to keep in the analysis only those that attended to 3 follow-ups (at 12, 24 and 48 months of age). Estimates were carried out using the Panel Data Tobit Model with random effects.

**Results:**

The study showed that expenditure on medicines was 20 % less likely in those considered healthy children by their mothers and, if there was any expenditure with healthy children, the expected expenditure was reduced by 58 %. A 1 % increase in household income increased the expected expenditure on medicines by 16 %, and by 23 % in children with private health insurance coverage.

**Conclusions:**

All types of health care expenditures examined were higher for children covered by private health insurance. Although total health care expenditure was higher for children of better-off families, it represented a lower share of these families’ income evidencing income inequality in health care expenditures.

**Electronic supplementary material:**

The online version of this article (doi:10.1186/s12939-015-0180-0) contains supplementary material, which is available to authorized users.

## Background

The study of determinants of health care expenditure has gained momentum since the 1970s with significant increases in health care expenditure as a percent of gross domestic product (GDP) among developed countries [1, 2].

According to the World Health Organization (WHO) Brazil’s health care expenditure accounted for 9 % of GDP in 2009, with only 45.7 % government-paid care costs. Contrasting data from the Brazilian Ministry of Health in 2003 showed that nearly 75 % of the Brazilian population relied on public health care [3].

Health care expenditures in Brazil are mostly on medicine and monthly premium payments of private health plans [4]. Although total health care costs in better-off families are higher, they represent a smaller share of these families’ income evidencing the regressive nature of family health expenditure [5]. Family health expenditure is much higher in families with children and elderly members [6]. Education level of the head of the family and per capita family income are key determinants of both the probability of health care expenditure and the amount spent [7].

International studies have shown that health care expenditure is sensitive to family income [7–11], and this correlation is even stronger in low-income families with no health insurance [9]. Factors such as income, education level, and being employed status negatively affect the probability of hospital care expenditure but this probability increases when there are in the family children under one, smokers, and members with any chronic condition [12].

Health care expenditure for children has been little explored in Brazil to date. Studies in US children have shown that 8 % of total expenditure whit them is health-related [13].

The present study aimed to examine the determinants of private expenditure on health care for children and to assess socioeconomic, anthropometric, and demographic factors associated.

## Methods

This study was based on data from the 2004 Pelotas Birth Cohort conducted by the Center for Epidemiologic Research at the Federal University of Pelotas, southern Brazil. This cohort enrolled live births in the city of Pelotas and a neighboring area of Jardim América district in the municipality of Capão do Leão in 2004. Pelotas had 328,275 inhabitants with a per capita GDP of BRL 10,734.00 according to the 2010 Population Census (IBGE, Brazilian institute of geography and statistics).

Of 4263 live births in the urban area in 2004, 4231 were included in the perinatal cohort study. Mothers were invited to participate and those who agreed were interviewed using a questionnaire and their children underwent newborn evaluation including measures of length, chest, and abdominal circumference [14].

Children have been followed up at six different points in time; first at baseline when a perinatal interview was conducted, then at 3, 12, 24, and 48 months, and 6–7 years of age. However, this analysis was based only on data from the 12-, 24-, and 48-month follow-up including 94 %, 93 %, and 92 % of the initial cohort, respectively, totaling 3799 children [15]. After adjustments for balanced panel data there remained a total of 2436 observations (children).

Estimates were carried out using the Panel Data Tobit Model with random effects. This choice was made because the analysis of expenditures particularly includes individuals with no expenditure on health care, and the sample comprises a combination of strictly positive and zero values. In this scenario simple estimation with the use of the Ordinary Least Squares (OLS) method cannot generate consistent parameter estimates because the censored sample is not representative of the population. Yet, a random effects model was used as a fixed effects model may produce inconsistent estimators in analyses where individual effects are not uniform [16]. A multilevel model analysis was performed and 2436 observations from the three follow-ups were included in a single data structure.

The panel analysis allows to controlling for individual heterogeneity and detecting and measuring more accurately external effects, making it more suitable to the study of adjustment dynamics [17].

The model results were analyzed in two different ways; first, the marginal effect of independent variables on the probability of expenditure was interpreted based on the individual impact of each variable while controlling for all other variables; second, the marginal effect of these independent variables was individually measured against the expected expenditure.

Monetary variables using logarithms were used for estimates following a theoretical assumption where results are interpreted in terms of elasticity [18].

As dependent variables, five types of health care expenditures, in the 30 days previous to the interview, were explored: expenditure on medicines; medical care expenditure; expenditure on laboratory tests and x-rays; other health-related expenditure; and private health insurance expenditure (monthly premiums). Since ‘other health-related expenditure’ was almost negligible it was excluded from the analysis.

The independent variables included children characteristics such as current weight and birth weight (standardized by mean deviation), number of hospitalizations after delivery, child health status reported by the mother (children were considered healthy when they reported either “good,” “very good,” or “excellent” health), and having private health insurance; household characteristics such as number of people living in the household, family income (the natural log of the sum of incomes of all household members); head of household (father or mother); maternal characteristics including age (with a quadratic term to accommodate for possible nonlinear changes); maternal self-reported health status (healthy when they reported either “good,” “very good,” or “excellent”), and maternal level of education at the time of the child birth.

The study protocol of the 2004 Pelotas birth cohort was approved by the Medical Ethics Committee of the Federal University of Pelotas, affiliated with the Brazilian Federal Medical Council.

## Results

There were a high proportion of families with no expenditure on health care making average expenditure conditional on positive values significantly higher than average direct expenditure. It reflects more accurately average expenditure on purchased services and products. The proportion of families with no expenditure on health care for children was 34.48 % at 12 months, 37.19 % at 24 months, and 39.86 % at 48 months of follow-up.

Table 1 shows that expenditure on medicines was the most common type of expenditure at the three follow-ups (48.15 % at 12 months, 45.20 % at 24 months, and 40.89 % at 48 months), followed by private health insurance expenditure. The latter was higher for older children (24.75 % at 12 months, 26.31 % at 24 months, and 27.34 % at 48 months). Medical care expenditure was higher for younger children (13.17 % at 12 months, 8.54 % at 24 months, and 7,72 % at 48 months).

**Table 1:** Average expenditure (BRL^a^), percentage of families with expenditure and average conditional expenditure (BRL) for five types of health care expenditure for children. The 2004 Pelotas Birth Cohort. Pelotas, Brazil

Variable	Follow-up (months)	Average expenditure (BRL) E(x)	% families with expenditure	Average conditional expenditure (BRL)
		*N* = 2436	*N* = 2436	E(x) | x > 0
Expenditure on medicines				
	12	18.18	48.15 %	37.75 (*N* = 1173)
	24	16.54	45.20 %	36.59 (*N* = 1101)
	48	14.47	40.89 %	35.39 (*N* = 996)
Medical care expenditure				
	12	4.85	13.17 %	36.80 (*N* = 321)
	24	3.36	8.54 %	39.35 (*N* = 208)
	48	3.18	7.72 %	41.20 (*N* = 188)
Expenditure on laboratory tests and x-rays				
	12	1.37	4.43 %	30.90 (*N* = 108)
	24	0.97	2.63 %	36.92 (*N* = 64)
	48	0.77	3.08 %	25.01 (*N* = 75)
Other health-related expenditure				
	12	0.30	0.33 %	91.35 (*N* = 8)
	24	0.46	0.29 %	160.08 (*N* = 7)
	48	0.86	0.94 %	91.08 (*N* = 23)
Private health insurance expenditure				
	12	17.63	24.75 %	71.22 (*N* = 603)
	24	17.25	26.31 %	65.55 (*N* = 641)
	48	17.32	27.34 %	63.35 (*N* = 666)

^a^BRL = Brazilian *real.* Exchange rate: 1.00 USD = 2.40 BRL (December 2008)

The mean incomes in each follow-up (whole sample, 1st decile, 10th decile) are, respectively, in BRL: 12 months (1,121.62, 179.27, 4,401.45); 24 months (1,248.29, 191.28, 4,976.89); 48 months (1,468.27, 277.23, 5,677.82) (Additional file 1).

**Fig. 1:**
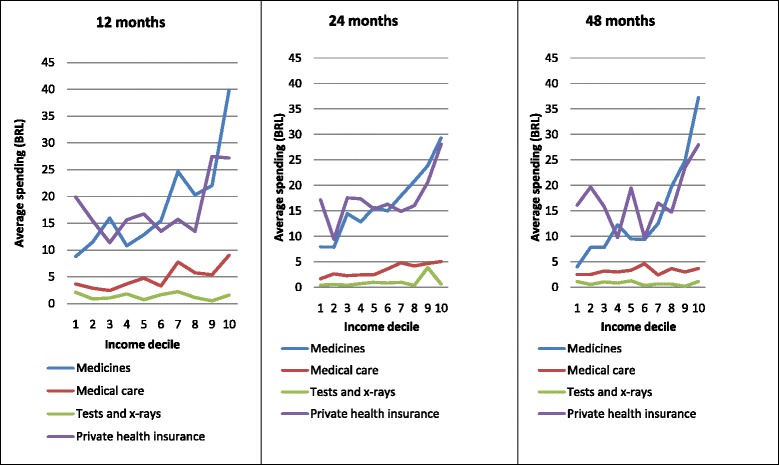
Average expenditure on health care for children by household income decile at 12, 24, and 48 months of follow-up. The 2004 Pelotas Birth Cohort. Pelotas, Brazil (*N* = 2436)

Figure 1 shows average health care expenditure stratified by household income deciles. At the 12-month follow-up, average expenditure increased with household income (except for expenditure on laboratory tests and x-rays that was almost insignificant). This same trend was seen at 24 and 48 months of follow-up.

**Fig. 2:**
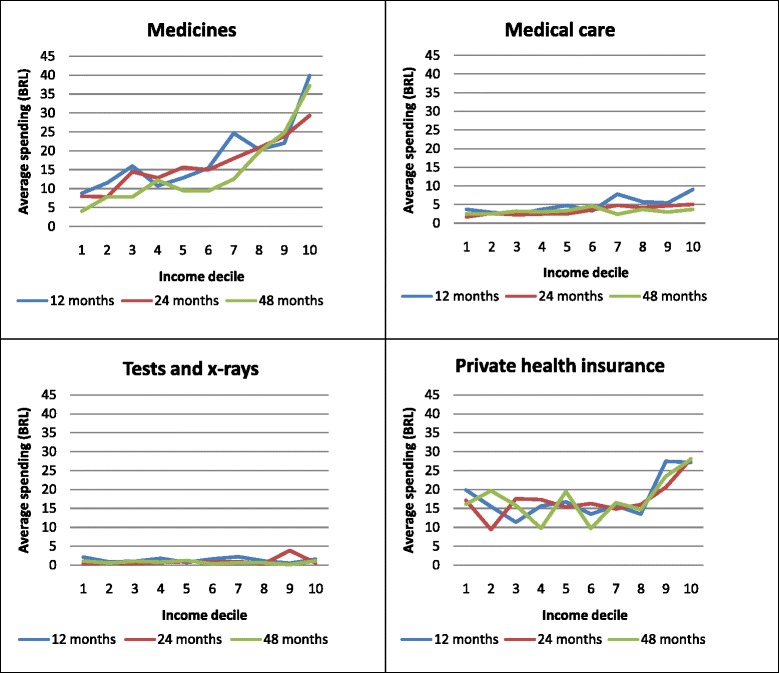
Trends of average expenditure on health care for children by household income deciles and type of expenditure at 12, 24 and 48 months of follow-up. The 2004 Pelotas Birth Cohort. Pelotas, Brazil (*N* = 2436)

Expenditure on medicines trend (Fig. 2) indicates that expenditure increases with household income deciles and decreases with child’s growth, i.e., average expenditure was higher for younger children. Higher medical care expenditure was seen at the 12-month than 24- and 48-month follow-up. It was also higher in upper income deciles at the three follow-ups analyzed. Expenditure on laboratory tests and x-rays did not show any significant differences across income deciles while private health insurance expenditure though inconsistent was higher in upper income deciles at the three follow-ups.

**Fig. 3:**
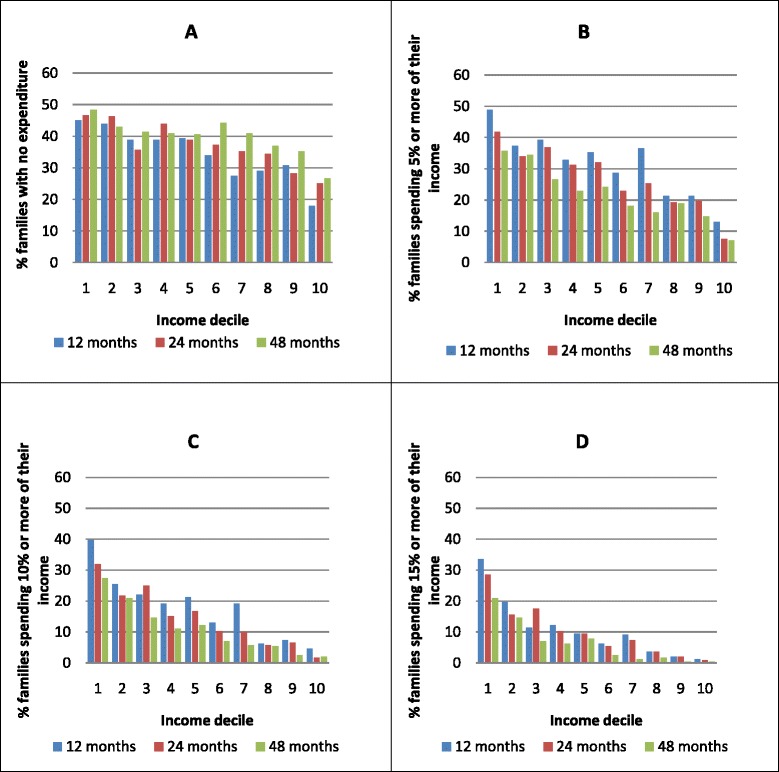
Percentage of families with no expenditure (0 %) **a**, expenditure 5 % or more **b**, 10 % or more **c**, and 15 % or more **d** on health care for their children. The 2004 Pelotas Birth Cohort. Pelotas, Brazil (*N* = 2436)

Figure 3 illustrates health care expenditure on families as a percentage of income. At the three follow-ups a large share of family income was spent on health care for children in a high proportion of families in lower income deciles. At the 12-month follow-up, nearly 34 % of poor families spent 15 % or more of their income on health care (Fig. 3d). At the 48-month follow-up a lower share of families’ income was spent on health care compared to the two previous follow-ups. The average proportion of families expending 15 % or more of their income on health care was 10.88 % at 12 months, 10.10 % at 24 months, and 6.28 % at 48 months of follow-up. It was also found a larger proportion of families with no health care expenditure among the poor (Fig. 3a).

Table 2 presents estimate results showing the effect of independent variables on the probability of health care expenditure. As for expenditure on medicines, almost all variables showed significant marginal effects at the level of 1 % in the analysis, and being a ‘healthy’ mother was significant at the level of 5 %. Being a ‘healthy’ child reduced by almost 20 % the probability of any expenditure on medicines while having private health insurance increased it by almost 8 %. A 1 % increase in household income increased by 5 % the probability of any health care expenditure for children.

**Table 2:** Marginal effect of independent variables on the probability of expenditure on health care for children (N = 7308). The 2004 Pelotas Birth Cohort. Pelotas, Brazil

	Marginal effect – Probability			
	Medicine	Medical care	Laboratory tests and x-rays	Private health insurance
Family income	0.0551^a^	0.0120^b^	−0.0014 +	0.0047 +
Maternal education (at child’s birth)	0.0136^a^	0.0034^b^	0.0006 +	0.0057^b^
Number of hospital visits	0.0490^a^	- 0.0050 +	0.0008 +	0.0073 +
Number of people living in the household	−0.0201^a^	−0.0055^b^	−0.0010 +	−0.0001 +
Maternal age	0.0189^a^	0.0014 +	−0.0048^b^	0.0060 +
Squared maternal age	−0.0003^a^	−0.0001 +	0.0001^b^	−0.0001 +
Child’s weight	−0.0078^a^	−0.0042^a^	−0.0008 +	0.0020^a^
Head of household – father	−0.0111 +	0.0122 +	0.0106 +	−0.0183 +
Head of household – mother	−0.0393^c^	−0.0020 +	0.0110 +	0.0057+
Private health insurance	0.0791^a^	0.0207^b^	0.0095^c^	-
Healthy mother	−0.0292^b^	−0.0121 +	−0.0099^c^	−0.0135 +
Healthy child	−0.1972^a^	−0.0239^c^	−0.0029 +	−0.0051 +
Birth weight (standardized)	0.0105^c^	−0.0011 +	−0.0010 +	0.0091 +

Total of 7308 observations; 2436 observations over the three follow-ups examined (2436 × 3)

Significance achieved with a z-test

^a^Indicates significance at 1 %; ^b^at 5 %; ^c^at 10 %; and + indicates the variable is not significant even at 10 %

A major variable associated with medical care expenditure was the child’s weight. However, despite being significant this variable produced less than 1 % increase in the probability of expenditure. Having private health insurance showed a significant marginal effect at the level of 5 % on the probability of medical care expenditure. A 1 % increase in family income increased the probability of expenditure by only 1 %.

Table 3 shows that the marginal effect of independent variables on the expected expenditure was conditional on positive values, i.e., when there was actually any expenditure. A 1 % increase in household income increased the expected expenditure on medicines by 16 %. Having private health insurance had a marked effect as it increased the expected expenditure on medicines by approximately 23 % and medical care expenditure by 12 %. And being a ‘healthy’ child reduced the expected expenditure on medicines by approximately 58 %.

**Table 3:** Marginal effect of independent variables on the expected expenditure conditional on positive values (*N* = 7308). The 2004 Pelotas Birth Cohort. Pelotas, Brazil

	Marginal effect – Expected conditional expenditure			
	Medicine	Medical care	Laboratory tests and x-rays	Private health insurance
Family income	0.1618^a^	0.0702^b^	−0.0164 +	0.0187 +
Maternal education (at child’s birth)	0.0399^a^	0.0200^b^	0.0071 +	0.0228^b^
Number of hospital visits	0.1439^a^	−0.0291 +	0.0094 +	0.0292 +
Number of people living in the household	−0.0590^a^	−0.0320^b^	−0.0107 +	−0.0001 +
Maternal age	0.0554^a^	0.0085 +	−0.0539^b^	0.0243 +
Squared maternal age	−0.0009^a^	−0.0001 +	0.0009^b^	−0.0004 +
Child’s weight	−0.0228^a^	−0.0243^a^	−0.0090 +	0.0079^a^
Head of household – father	−0.0327 +	0.0714 +	0.1201 +	−0.0737 +
Head of household – mother	−0.1154^c^	−0.0116 +	0.1252 +	0.0228 +
Private health insurance	0.2322^a^	0.1206^b^	0.1072^c^	-
Healthy mother	−0.0856^b^	−0.0706 +	−0.1122^c^	−0.0542 +
Healthy child	−0.5785^a^	−0.1393^c^	−0.0329 +	−0.0206 +
Birth weight (standardized)	0.0309^c^	−0.0064 +	−0.0116 +	0.0366 +

Total of 7308 observations; 2436 observations over the three follow-ups examined (2436 × 3)

Significance achieved with a z-test

^a^Indicates significance at 1 %; ^b^at 5 %; ^c^at 10 %; and + indicates the variable is not significant even at 10 %

## Discussion

Despite extensive literature on child health, few studies, both international and national, have focused on determinants of health care expenditure for children under five years of age [19, 20].

The finding that expenditure on medicines was the most common type of expenditure (found in more than 40 % of the families) at the three follow-ups analyzed is corroborated by previous studies [7, 20] with similar results. Yet, this study found a health care expenditure trend inversely proportional to the child’s growth. This finding is also explained by the fact that medicine use prevalence is also inversely proportional to child’s growth during early childhood [21].

Expenditure on medicines showed to be more sensitive to different income levels: it was four times higher at 12 and 24 months and 8 times higher at 48 months follow-up in upper than lower income deciles, but income varied 24, 25 and 20 times between deciles in the three follow-ups, respectively. This finding contrasts with earlier results showing that expenditure on medicines was inelastic to income changes [7].

Medical care expenditure is higher for younger children, which is perfectly justified since children at this stage of life require more medical care. This finding is consistent with that of an earlier study [22] that showed a trend of higher expenditure for younger children. The large number of families with no expenditure on health care – mostly for all types of expenditure – resulted in a significantly higher average expenditure conditional on positive values than the average expenditure estimated based on both families with expenditure and no expenditure. For medical care expenditure in particular there was found an inverse relationship between simple average and conditional average expenditure: the former decreased as the latter increased with child’s growth. In fact, the incidence of expenditure on health care is reduced as a child grows, and thus the denominator (number of families with any expenditure on health care) decreases making the average conditional expenditure higher.

The impact of income elasticity was more pronounced on expenditure on medicines. This is a concerning finding because medicines are purchased as they are necessary. It may suggest that poor families cannot afford to meet their children’s needs on health care. However, these families may have access to medicine provided through the public health care system [23].

Another major finding in this study is inequality among income deciles. At the 48-month follow-up, 7.79 % of children in the lower decile had private health insurance coverage compared to 82.50 % in the upper decile. It is consistent with other Brazilian study reporting health care expenditure is mostly on medicine among low-income families whereas it is mostly on monthly premiums for private health insurance among better-off families [4]. Thus, in case of need for care, better-off families can seek private care while the poor may get only public health care.

It is of note that maternal education is the second major variable, behind child’s weight, affecting private health insurance expenditure. Private health insurance expenditure is a precautionary one as a child needs to be insured to receive care when needed. Better educated mothers are more likely to safeguard the health of their children [24], and to ensure they will be able to afford care in the event of a serious health problem and/or increased health care needs. However, the effect on the probability of expenditure is low.

The study also found that families in lower income deciles pay a larger share of their incomes toward health care for children than do families in higher ones, a finding that is corroborated in the literature regarding expenditure on health care [25] and medicines [23], and condition-related expenditure [26]. At the 48-month follow-up, a smaller proportion of families spent a substantial amount of their income on health care, which can be explained by either a marked increase in real wages during the study period or actually less expenditure as a child grows. Regarding health catastrophic expenditure for children only, the average percentage of families that spent 15 % or more of their income on child health care was 10.88 % at 12 months, 10.10 % at 24 months, and 6.28 % at 48 months of follow-up. However, this analysis may vary depending on the definition of catastrophic health expenditure used in studies. Catastrophic health expenditure is defined as expenditure in excess of 40 % of the household’s capacity to pay (household non-subsistence effective income) [27], or the percentage of total income spent on care above a certain threshold (10 %, 15 %, or 20 %) [28]. It is noteworthy that a high proportion of families were expending more than 15 % of their income on health care for their children.

Our study has some limitations. First, the child health status reported by the mother is not an objective measure of health. Because that measure depends on the mother’s perception, it is assumed that she comprehends the categories of response as she reports on the child health, therefore there are no measurement errors. However, the subjective measures are not perfect and there may be a difference between what the mother perceives to be the child health and the actual child health. This difference may be due to variations in mother’s culture, knowledge, beliefs and information. One way of minimizing this limitation in the present study was the use of objective measures such as the current weight of the child and the birth weight.

Second, a factor not addressed in our analysis is the possible reverse causality between out-of-pocket expenditure on health care for children and child health. The child health status can affect the expenses incurred for the health care for children, requiring medicines, medical care, laboratory tests and X-rays, private health insurance, and other health-related expenditure, which is what was analyzed in this study. At the same time, the expenses paid for health care for child may also affect the child health status. It is hoped that along with money spent on medicines, medical care, laboratory tests and X-rays, private health insurance, and other health-related expenditure, one can anticipate and avoid a worsening of the child health status. In this sense, we would have a relationship of causality in both directions: health expenditures affecting health, and health affecting health expenditures.

## Conclusions

The study results showed that the probability of expenditure on medicines for children was strongly affected by family socioeconomic characteristics, as well as maternal education level (at the child’s birth), and evidently by the child’s health status. Expenditure on medicines was almost 20 % less likely in those considered ‘healthy’ children and almost 8 % more likely in those with private health insurance coverage. Our results agree with those found in the literature [7] showing that health care expenditure is more likely among families with higher per capita income and higher education level of the head of household.

In the light of that, public policies should be developed and implemented to ensure proper functioning of public health services so that low-income children can get any medical care they need without imposing further financial burden on their families. It allows these families to allocate their budget to other important aspects of their children’s life, such as food, education, leisure time, without compromising their health care.

## Additional file

Additional file 1:
**Mean incomes and minimum values (BRL** ^**1**^ **) in each follow-up, according to income deciles.** The 2004 Pelotas Birth Cohort. Pelotas, Brazil.
