# Supplementary material for: Determinants of out-of-pocket health expenditure on children: an analysis of the 2004 Pelotas Birth Cohort
Source: Int J Equity Health. 2015 Jun 9;14:53. doi: 10.1186/s12939-015-0180-0 (PMC4467315; doi:10.1186/s12939-015-0180-0)
Supplement: Additional file 1: — Mean incomes and minimum values (BRL 1 ) in each follow-up, according to income deciles. The 2004 Pelotas Birth Cohort. Pelotas, Brazil. [file 12939_2015_180_MOESM1_ESM.docx]

**Additional file 1**: Mean incomes and minimum values (BRL^1^) in each follow-up, according to income deciles. The 2004 Pelotas Birth Cohort. Pelotas, Brazil

| Family income | | | | | | |
| --- | --- | --- | --- | --- | --- | --- |
|  | **12 months** | | **24 months** | | **48 months** | |
| Decil | **Minimum** | **Average** | **Minimum** | **Average** | **Minimun** | **Average** |
| 1st | 17.57 | 179.27 | 16.77 | 191.28 | 41.38 | 277.23 |
| 2nd | 291.43 | 343.23 | 328.03 | 379.97 | 416.49 | 468.43 |
| 3rd | 388.57 | 435.16 | 433.92 | 469.63 | 521.70 | 579.04 |
| 4th | 472.55 | 531.19 | 515.42 | 577.85 | 635.11 | 720.47 |
| 5th | 586.49 | 651.18 | 654.15 | 703.53 | 808.59 | 874.97 |
| 6th | 701.27 | 771.16 | 782.91 | 854.98 | 955.76 | 1,036.19 |
| 7th | 850.59 | 961.76 | 934.64 | 1,056.31 | 1,134.07 | 1,257.15 |
| 8th | 1,070.26 | 1,230.33 | 1,176.59 | 1,357.25 | 1,406.32 | 1,597.10 |
| 9th | 1,410.81 | 1,725.86 | 1,570.35 | 1,928.59 | 1,854.93 | 2,239.53 |
| 10th | 2,205.58 | 4,401.35 | 2,438.56 | 4,976.89 | 2,779.53 | 5,677.82 |
| Total |  | 1,121.62 |  | 1,248.29 |  | 1,468.27 |

^1^BRL = Brazilian *real.* Exchange rate: 1.00 USD = 2.40 BRL (December 2008).
